# Relationship between red cell storage duration and outcomes in adults receiving red cell transfusions: a systematic review

**DOI:** 10.1186/cc12600

**Published:** 2013-04-08

**Authors:** Christophe Lelubre, Jean-Louis Vincent

**Affiliations:** 1 Department of Intensive Care, Erasme Hospital, Université Libre de Bruxelles, Route de Lennik 808, 1070 Brussels, Belgium

## Abstract

**Introduction:**

The duration of red blood cell (RBC) storage before transfusion may alter RBC function and supernatant and, therefore, influence the incidence of complications or even mortality.

**Methods:**

A MEDLINE search from 1983 to December 2012 was performed to identify studies reporting age of transfused RBCs and mortality or morbidity in adult patients.

**Results:**

Fifty-five studies were identified; most were single-center (93%) and retrospective (64%), with only a few, small randomized studies (eight studies, 14.5%). The numbers of subjects included ranged from eight to 364,037. Morbidity outcomes included hospital and intensive care unit (ICU) length of stay (LOS), infections, multiple organ failure, microcirculatory alterations, cancer recurrence, thrombosis, bleeding, vasospasm after subarachnoid hemorrhage, and cognitive dysfunction. Overall, half of the studies showed no deleterious effects of aged compared to fresh blood on any endpoint. Eleven of twenty-two (50%) studies reported no increased mortality, three of nine (33%) showed no increased LOS with older RBCs and eight of twelve (66%) studies showed no increased risks of organ failure. Ten of eighteen (55%) studies showed increased infections with transfusion of older RBCs. The considerable heterogeneity among studies and numerous methodological flaws precluded a formal meta-analysis.

**Conclusions:**

In this systematic review, we could find no definitive argument to support the superiority of fresh over older RBCs for transfusion.

## Introduction

Red blood cell (RBC) transfusions are one of the most common medical interventions [1]. Although they can be lifesaving, RBC transfusions have come under intense scrutiny over the last few decades [2]. In a recent review of 45 observational studies that had reported the impact of transfusion on outcomes in different patient groups (trauma; general, cardiac, orthopedic and neurosurgical; acute coronary syndrome; intensive care (ICU) patients), RBC transfusion was an independent predictor of death, infectious complications and acute respiratory distress syndrome (ARDS) [3].

The 'storage lesion' refers to the multiple complex biochemical and biomechanical alterations that occur during *ex vivo *storage, modifying RBC properties and the supernatant [4] (Table 1). A progressive decrease in 2,3-diphosphoglycerate (DPG) levels may impair oxygen delivery [5], and a decreased adenosine triphosphate (ATP) pool reduces Na^+^-K^+^-ATPase activity and, possibly, ATP-mediated hypoxic vasodilation. Decreased antioxidant capacities of the erythrocyte may alter reduction of methemoglobin [6], generating reactive oxygen species (ROS) through the Fenton reaction. Complex, irreversible membrane changes (including alterations in protein band 3 [7] and release of procoagulant [8] vesicles) lead to poorly deformable sphero-echinocytes with increased adherence to the endothelium [9] and increased susceptibility to phagocytosis [10]. Changes also occur in the supernatant, with a progressive decrease in pH, increased potassium concentration, and release of proinflammatory molecules, complement or biologically active lipids (platelet-activating factor). Release of hemoglobin (free or contained in microparticles) may scavenge nitric oxide (NO) in the transfusion recipient and lead to vasoconstriction [11] and iron and heme may generate redox injuries, cytotoxicity and inflammation [12,13]. RBCs prone to increased oxidative injury undergo protein and lipid peroxidation with release of lysophospholipids, which may cause transfusion-associated acute lung injury (ALI) [14]. However, animal models describing altered blood flow following transfusion of old RBCs [15,16] may be of limited relevance to humans [17]. Some alterations described *ex vivo*, such as the decreases in 2,3-DPG or ATP levels, may be at least partly reversible after transfusion [17]. Therefore, the impact of storage lesions observed *in vitro *may not be relevant clinically [18,19]. Some recent meta-analyses [20-22] have been attempted, but the heterogeneity and methodological limitations of the studies included prevented definitive conclusions. The duration of RBC storage varies among units. Large observational studies have reported a mean duration of RBC storage of between 16 and 21 days [23], with a maximum storage duration before transfusion being generally limited to 42 days (with standard preservative solutions). However, the regulatory requirements for this limit are based only on the percentage RBC survival 24 h after transfusion (which has to be ≥ 75%), not on oxygen delivery capacities or clinical endpoints [24].

**Table 1:** Main components of the storage lesion.

Changes occurring to the RBC	Changes occurring in the supernatant
*Metabolic changes*	
	
• Decreased 2-3 DPG, possibly with impaired oxygen delivery [5] • Decreased phosphate and adenine pool (AMP, ADP, ATP) [24] • Decreased glutathione levels [6] • Decreased S-nitroso hemoglobin [110] • Increased lactate levels	• Decreased pH • Increased potassium concentrations (decreased Na-K-ATPase activity) with increased risks of hyperkalemia • Release of various molecules: ○ Proinflammatory cytokines (IL-1beta, IL-6, IL-8, TNF-alpha) and complement ○ Biologically active lipids such as platelet-activating factor (PAF) [14] ○ Free hemoglobin prone to scavenge nitric oxide (NO) of the recipient (together with Hb-containing microparticles) [109] ○ Heme and iron [12] with potential redox injuries, cytotoxicity and inflammation
	
*Oxidative stress*	
	
• Protein oxidation including cytoskeleton [111] • Lipid peroxidation, generation of lysophospholipids prone to cause cases of TRALI, generation of prostaglandins and isoprostanes [112]	
	
*Shape and membrane changes*	
	
• Shift from early reversible echinocytes to irreversible sphero-echinocytes • Generation of microvesicles with procoagulant properties • Increased RBC rigidity and adherence to vascular endothelium^2^ • Decreased CD47 expression, increased phosphatidylserine exposure	

ADP, adenosine diphosphate; AMP, adenosine monophosphate; ATP, adenosine triphosphate; DPG, diphosphoglycerate; Hb, hemoglobin; IL, interleukin; RBC, red blood cell; TNF-alpha, tumor necrosis factor alpha; TRALI, transfusion-related acute lung injury.

The aim of this article was to review the clinical evidence related to the potential impact of RBC storage on outcomes in adult patients.

## Materials and methods

**Figure 1:**
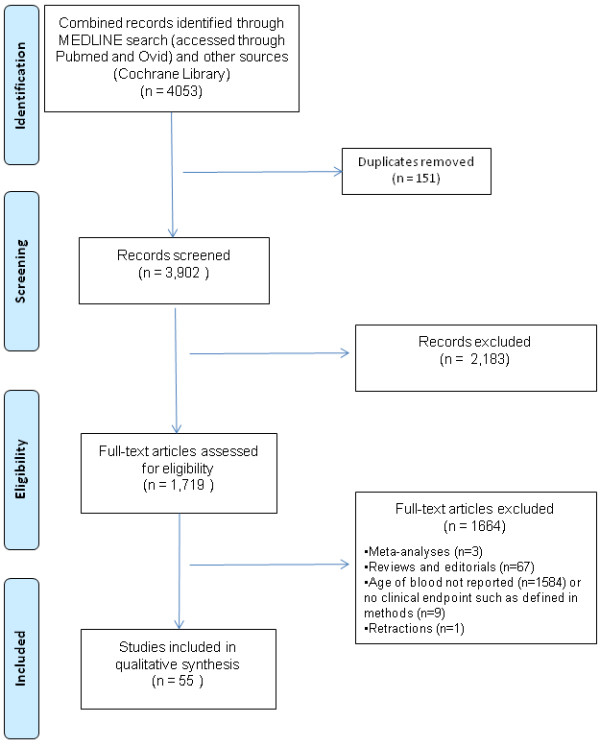
**Flow diagram of study selection**.

A MEDLINE search (accessed through Pubmed and Ovid) of the literature was performed to identify studies in adult patients that linked the duration of storage of transfused RBCs with physiologic variables (oxygen consumption, alterations of microcirculation), morbidity and mortality. Morbidity outcomes included infection; postoperative bleeding; thrombosis, including myocardial infarction; duration of mechanical ventilation; multiple organ dysfunction including acute renal failure; ICU and hospital lengths of stay (LOS); and other less common endpoints, such as cancer recurrence, vasospasm after subarachnoid hemorrhage (SAH) or cognitive dysfunction. Medical Subject Headings (MeSH) terms ('Erythrocytes', 'Blood Transfusion', 'Mortality', 'Infection', 'Pneumonia', 'Acute Kidney Injury', 'Length of Stay', 'Multiple Organ Failure', 'Respiration, Artificial', 'Venous Thrombosis', 'Neoplasms', 'Microcirculation', 'Oxygen Consumption', 'Vasospasm, Intracranial') and non-MeSH terms ('Age', 'Storage', 'Duration') were used for the search process. All studies on adult patients (more than 19 years old) published from 1983 to December 2012 (last access, December 15, 2012) and written in any language were selected for individual screening. The Cochrane Library was searched using the same search strategy. Bibliographic references of relevant studies were also checked manually to complete the search process. Review articles, editorials, meta-analyses, abstracts of scientific meetings or studies that were subsequently retracted were not included. The flow diagram related to the search process is shown in Figure 1 according to the PRISMA statement [25]. Once selected, additional data regarding potential confounders (for example, data on leukoreduction status) were collected, and studies were grouped according to their primary outcome measure for qualitative synthesis. Given their particular importance in causal inference, randomized controlled trials (RCTs) were also considered separately.

Mean values are given +/- standard deviation (SD) and median values are given with interquartile ranges (IQR). All relative risks (RR), odds ratios (OR) or hazard ratios (HR) are provided with 95% confidence intervals (CI).

## Results

The search process retrieved 4,053 (3,902 after duplicate removal) studies, of which 2,183 were excluded, leaving 1,719 studies that were assessed for eligibility (Figure 1). Of these, 1,664 were excluded for various reasons (reviews and editorials, meta-analyses, subsequent retraction of one study on cardiac surgery patients [26,27], and studies not reporting RBC storage variables or no clinical endpoint as defined earlier). Accordingly, the analysis included 55 studies (Table 2), with a total of 407,185 patients. Most studies were retrospective (*n *= 35, 64%) and single-center (*n *= 51, 93%). The number of transfused subjects ranged from eight to 364,037 (median: 268 patients, IQR: 61 to 757). Only a small proportion of the studies were randomized trials (*n *= 8, 14.5%); the eight randomized studies had fewer included patients (median = 21 patients, IQR: 10.5 to 83.75) and, in the majority, physiological variables were reported as the primary outcome measure (*n *= 6 studies) (Table 3).

**Table 2:** Overview of the studies reporting the age of transfused red blood cells (RBCs) and patient outcomes.

First author	Year	Year(s) of enrollment	Design	Country/continent	Leuko-reduction	Setting	Number of patients transfused	Summary of main results
**Studies addressing mortality**								

Purdy *et al. *[42]	1997	1992	Retrospective single-center	Canada	NR	ICU septic	31	Non-survivors received a greater proportion of older RBCs

Mynster *et al. *[53]	2001	1991-1993	Retrospective multicenter	Europe	No	Cancer	740	Mortality not different between patients transfused with RBCs aged more or less than 21 days

Murrell *et al. *[48]	2005	2001-2003	Retrospective single-center	USA	Mix (95% LR)	Trauma	275	No correlation between 'dose of aged blood' and hospital mortality

van de Watering *et al. *[38]	2006	1993-1999	Retrospective single-center	Europe	No	Cardiac surgery	2,732	No correlation between storage time variables and 30-day mortality

Koch *et al. *[32]	2008	1998-2006	Retrospective single-center	USA	Mix	Cardiac surgery	6,002	Higher hospital and one-year mortality rate in older blood group

Yap *et al. *[41]	2008	2001-2007	Retrospective single-center	Australia	Mix (3.8% LR)	Cardiac surgery	670	No association between storage duration and mortality

Dessertaine *et al. *[43]	2008	2005-2006	Retrospective single-center	Europe	Yes	ICU	534	No association between the age of transfused red cells and mortality

Weinberg *et al. *[44]	2008	2000-2007	Retrospective single-center	USA	Yes	Trauma	1,813	Transfusion of large (but not small) volumes of older blood associated with an increased risk of death

Weinberg *et al. *[46]	2008	2000-2007	Retrospective single-center	USA	Yes	Trauma	430	Slightly increased mortality with transfusion of older blood (> 14 days old)

Spinella *et al. *[47]	2009	2004-2007	Retrospective single-center	USA	Mix	Trauma	202	Hospital mortality higher for patients transfused with older RBCs; increased storage time independently associated with mortality

Edgren *et al. *[30]	2010	1995-2002	Retrospective multicenter	Europe	Mix	Mix	364,037	5% increased mortality after two years of follow-up

Weinberg *et al. *[45]	2010	2000-2009	Retrospective single-center	USA	Yes	Trauma	1,647	Trend toward higher mortality rate with older blood when transfused more than three RBC units

Eikelboom *et al. *[50]	2010	2002-2006	Retrospective single-center	Canada	Yes	Cardiovascular disease	4,933	Greater risk of hospital mortality in the highest age quartile of RBCs

Robinson *et al. *[51]	2010	1999-2005	Retrospective single-center	Canada	NR	PCI	909	Storage duration associated with 30-day mortality

van Straten *et al. *[40]	2011	1998-2007	Retrospective single-center	Europe	Yes	Cardiac surgery	3,597	No effect of RBC storage duration on early or late postoperative mortality

McKenny *et al. *[39]	2011	2002-2007	Retrospective single-center	Europe	Yes	Cardiac surgery	1,153	No association between RBC storage duration and postoperative mortality

Kekre *et al. *[54]	2011	2002-2007	Retrospective single-center	Canada	Yes	HSCT	555	Non-relapse 100-day mortality reduced in the subgroup of patients receiving exclusively old blood (> 14 days old)

Pettila *et al. *[29]	2011	2008	Prospective, multicenter	Australia - New Zealand	Mix	ICU	757	Exposure to the highest age quartile of RBCs associated with a higher hospital mortality rate compared to lowest quartile

Hassan *et al. *[49]	2011	2003-2005	Retrospective single-center	USA	NR	Trauma	820	Number of older units not associated with increased mortality

Saager *et al. *[31]	2012	2005-2009	Retrospective single-center	USA	Yes	Non-cardiac surgery	6,994	No relationship between median RBC storage duration and postoperative mortality (up to two years)

Dunn *et al. *[52]	2012	2000-2010	Retrospective single-center	USA	NR	Liver transplant	509	Transfusion of older blood not associated with increased two-year mortality

Kor *et al. *[28]	2012	2008-2010	Randomized single-center study	USA	Yes	ICU	100	Similar mortality between fresh and standard issue RBCs but not powered for this outcome

**Studies addressing ICU or hospital length of stay**								

Vamvakas *et al. *[56]	2000	1995	Retrospective single-center	USA	Mix (mostly not)	Cardiac surgery	268	No association between RBC storage duration and postoperative ICU and hospital LOS

Keller *et al. *[58]	2002	1998-1999	Retrospective single-center	USA	No	Trauma	86	Number of units of blood older than 14 days associated with an increased hospital LOS

Leal-Noval *et al. *[55]	2003	1998-2000	Prospective single-center	Europe	No	Cardiac surgery	585	No association between mean duration of RBC storage and ICU LOS

Murrell *et al. *[48]	2005	2001-2003	Retrospective single-center	USA	Mix (95% LR)	Trauma	275	Association between 'dose of aged blood' and ICU LOS

van de Watering *et al. *[38]	2006	1993-1999	Retrospective single-center	Europe	No	Cardiac surgery	2,732	No correlation between storage time variables and ICU LOS

Yap *et al. *[41]	2008	2001-2007	Retrospective single-center	Australia	Mix (3.8% LR)	Cardiac surgery	670	No association between length of storage and ICU LOS

McKenny *et al. *[39]	2011	2002-2007	Retrospective single-center	Europe	Yes	Cardiac surgery	1,153	No association between length of storage and ICU LOS

Sanders *et al. *[57]	2011	2005-2007	Retrospective single-center	Europe	Yes	Cardiac surgery	444	Age of blood was a significant but modest predictor of postoperative LOS

Kekre *et al. *[54]	2011	2002-2007	Retrospective single-center	Canada	Yes	HSCT	555	No correlation between age of transfused RBCs and hospital LOS

**Studies addressing age of transfused RBCs and occurrence of infections**								

Edna *et al. *[61]	1998	1980-1992	Retrospective single-center	Europe	No	Cancer	240	Age of RBCs not different in subjects developing postoperative infections and those who did not

Vamvakas *et al. *[59]	1999	1995	Retrospective single-center	USA	Mix	Cardiac surgery	256	Independent relationship between the age of transfused RBCs and the incidence of postoperative pneumonia or wound infection

Mynster *et al. *[53]	2001	1991-1993	Retrospective multicenter	Europe	No	Cancer	740	Increased risks of postoperative infections with transfusions of RBCs aged 20 days or more

Offner *et al. *[64]	2002	1992-	Retrospective single-center	USA	No	Trauma	61	Number of units older than 14 and 21 days as an independent risk factor for major infections

Leal-Noval *et al. *[55]	2003	1998-2000	Prospective single-center	Europe	No	Cardiac surgery	585	Independent relation between the oldest RBC unit and postoperative pneumonia

Taylor *et al. *[66]	2006	2001-2003	Prospective	USA	Mix	ICU	449	Maximum age of transfused RBCs not associated with increased risks of nosocomial infections

Koch *et al. *[32]	2008	1998-2006	Retrospective single-center	USA	Mix	Cardiac surgery	6,002	Higher rates of postoperative sepsis or septicemia (but not pneumonia or wound infections) with older RBCs

Weinberg *et al. *[46]	2008	2000-2007	Retrospective single-center	USA	Yes	Trauma	430	Occurrence of pneumonia related to the volume of old blood (> 14 days old) transfused

Yap *et al. *[41]	2008	2001-2007	Retrospective single-center	Australia	Mix (3.8% LR)	Cardiac surgery	670	No association between storage of RBCs and postoperative pneumonia

Dessertaine *et al. *[43]	2008	2005-2006	Retrospective single-center	Europe	Yes	ICU	534	No independent association between the age of RBCs and nosocomial infections

Vandromme *et al. *[63]	2009	2004-2007	Retrospective single-center	USA	Yes	Trauma	1,183	Increased risks of pneumonia after transfusion of exclusively old RBCs (> 14 days)

Basora *et al. *[113]	2010	2004-2005	Retrospective single-center	Europe	Yes	Knee arthroplasty	335	No independent association between age of transfused RBCs and postoperative wound infection

McKenny *et al. *[39]	2011	2002-2007	Retrospective single-center	Europe	Yes	Cardiac surgery	1,153	No association between storage of RBCs and postoperative pneumonia

Andreasen *et al. *[60]	2011	2003-2008	Retrospective multicenter	Europe	No	Cardiac surgery	1,748	Greater risk of postoperative wound infections and septicemia with RBCs older than 14 days

Hassan *et al. *[49]	2011	2003-2005	Retrospective single-center	USA	NR	Trauma	820	Number of units older than 14 days as a significant risk factor for severe sepsis or septic shock

Juffermans *et al. *[67]	2011	2004-2007	Retrospective single-center	Europe	Yes	ICU septic	67	Storage time as a confounder for the association of RBCs with infection

Juffermans *et al. *[65]	2012	2004-2007	Retrospective single-center	Europe	Yes	Trauma/TBI	196	Modest association between transfusion of RBCs older than 14 days and occurrence of new infections

Dunn *et al. *[52]	2012	2000-2010	Retrospective single-center	USA	NR	Liver transplant	509	No independent association between the age of RBCs and postoperative infections

**Studies addressing organ failure**								

Zallen *et al. *[68]	1999	NR	Retrospective single-center	USA	NR	Trauma	63	Mean age of blood (or number of units older than 14 or 21 days) as independent risk factors for MOF

Vamvakas *et al. *[56]	2000	1995	Retrospective single-center	USA	Mix (mostly not)	Cardiac surgery	268	Length of RBC storage not associated with prolonged endotracheal intubation

Keller *et al. *[58]	2002	1998-1999	Retrospective single-center	USA	No	Trauma	86	Duration of MV not associated with RBC storage duration

Leal-Noval *et al. *[55]	2003	1998-2000	Prospective single-center	Europe	No	Cardiac surgery	585	No relationship between RBC storage duration and prolonged MV

Gajic *et al. *[70]	2004	2001	Retrospective single-center	USA	Mix	ICU	181	No association between mean age or age of the oldest unit transfused and occurrence of ALI

Yap *et al. *[41]	2008	2001-2007	Retrospective single-center	Australia	Mix (3.8% LR)	Cardiac surgery	670	No association between storage duration and occurrence of postoperative renal failure or prolonged MV

Koch *et al. *[32]	2008	1998-2006	Retrospective single-center	USA	Mix	Cardiac surgery	6,002	Increased risk of a composite outcome (including MOF and renal failure) with transfusion of older blood; higher rates of MV > 72 hours when patients transfused only old RBC

Weinberg *et al. *[46]	2008	2000-2007	Retrospective single-center	USA	Yes	Trauma	430	Old blood (> 14 days) associated with acute renal dysfunction after adjustment

McKenny *et al. *[39]	2011	2002-2007	Retrospective single-center	Europe	Yes	Cardiac surgery	1,153	No association between storage duration and occurrence of postoperative renal failure or prolonged MV

Sanders *et al. *[57]	2011	2005-2007	Retrospective single-center	Europe	Yes	Cardiac surgery	444	Higher rates of renal failure among patients transfused with old blood

Kor *et al. *[28]	2012	2008-2010	Randomized single-center study	USA	Yes	ICU	100	Similar measures of pulmonary function after transfusion of either fresh or standard issue RBCs

Weiskopf *et al. *[69]	2012	NR	Randomized single-center study	USA	Mix	Volunteers	35	Equivalent mild decrements in pulmonary gas exchange variables after transfusion of either fresh (1.7 hour) or older (> 21 days) RBCs

**Studies addressing tissue oxygenation and microcirculation**								

Marik *et al. *[71]	1993	NR	Prospective single-center study	USA	NR	ICU	23	Inverse correlation between the age of transfused RBCs and the maximal change in gastric mucosal pH (pHi)

Fernandes *et al. *[72]	2001	1996	Randomized single-center study	South America	NR	ICU septic	15	No correlation between the age of transfused RBCs and the pHi

Walsh *et al. *[73]	2004	1999-2000	Randomized single-center study	Europe	Yes	ICU	22	No difference in pHi or gastric to arterial PCO_2 _gap with transfusions of fresh or older RBCs

Sakr *et al. *[75]	2007	NR	Prospective single-center	Europe	Yes	ICU	35	No correlation between the storage time and the changes in capillary perfusion

Leal-Noval *et al. *[77]	2008	2004-2006	Prospective single-center	Europe	Yes	Severe TBI	66	Transfusion of RBCs aged more than 19 days failed to increase cerebral tissue oxygenation

Kiraly *et al. *[79]	2009	NR	Prospective single-center	USA	NR	Trauma	32	Patients transfused with > 21 days old RBCs had a significant decrease in the area under the curve of tissue saturation (StO_2_)

Creteur *et al. *[81]	2009	NR	Prospective single-center	Europe	Yes	ICU	44	No association between RBC storage time and oxygenation variables (NIRS)

Yuruk *et al. *[76]	2012	NR	Randomized single-center study	Europe	Yes	Hematology patients	20	Increase in perfused vessel density similar in both RBC age groups

Berra *et al. *[82]	2012	2010	Randomized single-center study	USA	Yes	Healthy volunteers	9	Reactive hyperemia index unchanged after transfusion of 40-day stored blood compared with transfusion of three-day stored blood

Roberson *et al. *[83]	2012	NR	Prospective single-center	USA	Yes	Healthy volunteers	8	No effect of storage duration on tissue oxygen saturation and sublingual microcirculatory flow index

Kopterides *et al. *[84]	2012	2008-2011	Prospective single-center	Europe	Mix	ICU septic	37	No relationship between age of RBCs and change in lactate/pyruvate ratio (microdialysis)

**Other outcomes**								

Wasser *et al. *[87]	1989	NR	Randomized single-center study	Europe	No	Cardiac surgery	237	No differences in postoperative bleeding, coagulation tests, or transfusion requirements

Mynster *et al. *[53]	2001	1991-1993	Retrospective multicenter	Europe	No	Cancer	740	Higher recurrence rate of cancer in patients who received a transfusion of RBCs stored < 21 days

Leal-Noval *et al. *[55]	2003	1998-2000	Prospective single-center	Europe	No	Cardiac surgery	585	No association between duration of storage of RBCs and postoperative myocardial infarction

Weiskopf *et al. *[88]	2006	NR	Randomized single-center study	USA	No	Volunteers	9	Reversal of anemia-induced cognitive dysfunction similar after transfusion of fresh or stored RBCs

Spinella *et al. *[47]	2009	2004-2007	Retrospective single-center	USA	Mix	Trauma	202	Association between maximum age of RBCs (> 21 or 28 days) and deep vein thrombosis (DVT)

Naidech *et al. *[86]	2011	NR	Retrospective single-center	USA	NR	SAH	119	No association between age of RBCs and vasospasm or cerebral infarction

Katsios *et al. *[85]	2011	2001-2002	Prospective single-center	Canada	No	ICU	261	No association between the age of transfused RBCs and the occurrence of DVT

Cata *et al. *[89]	2011	1998-2007	Retrospective single-center	USA	NR	Prostate cancer	316	No association between the age of transfused RBCs and the five-year prostate cancer recurrence

DVT, deep vein thrombosis; HSCT, hematopoietic stem cell transplantation; ICU, intensive care unit; LOS, length of stay; LR, leukoreduced; MV, mechanical ventilation; NIRS, near-infrared spectroscopy; NR, not reported; PCI, percutaneous coronary intervention; SAH, subarachnoid hemorrhage: TBI, traumatic brain injury.

**Table 3:** Randomized controlled trials assessing the effects of red blood cell (RBC) storage duration on various outcomes (feasibility trials excluded).

First author	Year published (recruitment period)	Country/region	Leuko-reduction	Setting	Number of patients	Number of units transfused	Age of RBCs in each group	Outcome(s) reported	Summary of results
Wasser *et al. *[87]	1989 (NR)	Europe	No	Cardiac surgery	237 (118 in fresh group, 119 in old group)	5.4 +/-1.9 (fresh group) and 6.0+/-2.4 (older group)	< 12 h (fresh group) vs. 2-5 days (older group)	Postoperative bleeding	No differences in postoperative bleeding, coagulation tests, or transfusion requirements

Fernandes *et al. *[72]	2001 (1996)	South America	NR	ICU septic patients	15 (10 transfused, 5 receiving 5% albumin)	1 unit	12.8 ± 8.1 days in transfused group	Oxygen delivery and consumption; changes in pHi (tonometry)	No correlation between the age of transfused RBCs and the pHi

Walsh *et al. *[73]	2004 (1999-2000)	Europe	Yes	ICU patients with MV	22 (10 in fresh group, 22 in old group)	2 units	2 days vs. 28 days (median durations)	pHi or gastric to arterial PCO_2 _gap	No difference in pHi or gastric to arterial PCO_2 _gap between fresh or older RBCs

Weiskopf *et al. *[88]	2006 (NR)	USA	No	Healthy volunteers	9 (crossover design)	2 autologous units	3.4 hours vs. 23 days (median durations)	Cognitive dysfunction	Reversal of anemia-induced cognitive dysfunction similar after transfusion of fresh or stored RBCs

Berra *et al. *[82]	2012 (2010)	USA	Yes	Healthy volunteers	9 (crossover design)	1 autologous unit	3 days vs. 40 days	Reactive hyperhemia index	Reactive hyperemia index unchanged after transfusion of 40-day stored blood compared with transfusion of three-day stored blood

Yuruk *et al. *[76]	2012 (NR)	Europe	Yes	Hematology patients	20 (10 per arm)	3 units	7 days vs. 23 days (median durations)	Sublingual microcirculation variables	Increase in perfused vessel density similar in both RBC age groups

Weiskopf *et al. *[69]	2012 (NR)	USA	Mix (57% LR)	Healthy volunteers	35 (crossover design)	2 autologous units	1.7 hours vs. 24.5 days	Pulmonary function tests (A-aDO2, V_D_/V_T_))	Equivalent mild decrements in pulmonary gas exchange variables after transfusion of either fresh or older RBCs

Kor *et al. *[28]	2012 (2008-2010)	USA	Yes	ICU patients with MV	100 (50 per arm)	1 unit in each group	4 days vs. 26.5 days (median durations)	Pulmonary function tests; mortality (not powered for this)	Similar measures of pulmonary function (and mortality) after transfusion of either fresh or standard issue RBCs

ICU, intensive care unit; LR, leukoreduced; MV, mechanical ventilation; NR, not reported.

### Effect on mortality

We identified 22 studies that reported mortality as an endpoint, 20 of which were retrospective. Six studies focused on trauma patients, five on cardiac surgery patients, four on ICU patients and seven on mixed or other populations (patients with colorectal cancer, patients with cardiovascular disease or undergoing percutaneous coronary intervention, patients receiving hematopoietic stem cell transplantation or undergoing liver transplantation). Among these 22 studies, only one was a randomized trial, but this single-center study was small and not primarily powered for mortality [28]. In this trial conducted on 100 mechanically ventilated ICU patients, patients randomized to exclusively receive fresh RBCs (*n *= 50, median RBC age: 4.0 days) had similar mortality rates (36% in the fresh group vs. 40% in the standard issue group, *P *= 0.41) than those receiving standard RBCs (*n *= 50, median RBC age: 26.5 days) [28]. We identified one prospective, observational study, an Australian multicenter study of 757 critically ill patients (including a mix of medical and surgical patients) in which exposure to the highest age quartile of RBCs (average age: 17.6 days) was associated with a significantly higher hospital mortality rate compared to lowest quartile (average age: 7.5 days) RBCs, even after correction for disease severity (APACHE III score) and the number of units transfused (OR: 2.01 (1.07 to 3.77), *P *= 0.03) [29].

The largest study to date exploring the association between age of transfused RBCs and mortality comes from the Swedish/Danish SCANDAT database exploring 404,959 transfusion episodes in more than 300,000 (mainly trauma and surgical) patients from 1995 to 2002: administration of RBCs aged 30 to 42 days was associated with a 5% increased mortality (HR: 1.05; 95% CI, 1.02 to 1.08) after two years of follow-up [30], but the authors acknowledged that this risk could have been overestimated in view of a greater baseline risk in recipients of older units. In another retrospective, registry-based analysis of 6,994 surgical patients with comorbidities (ASA score more than III in 80% of patients; cardiac surgery patients excluded) receiving a median of two leukoreduced RBC units, Saager *et al*. found no relationship between median RBC storage duration and postoperative mortality (up to two years) in a Cox regression model (adjusting for ABO group and number of units transfused) [31].

Some studies included more homogeneous patient populations. In cardiac surgery patients, the largest study was a single-center, retrospective study of 6,002 patients undergoing coronary artery bypass graft (CABG), valve surgery or both. Patients transfused with older blood (median: 20 days) had greater hospital mortality compared to those receiving fresher (median: 11 days) blood (2.8% vs. 1.7%, *P *= 0.004). Patients transfused with older blood also had a higher one-year mortality rate (11.0 vs. 7.4%, *P *< 0.001) [32]. However, these results were much debated and challenged by other groups that were unable to find such an association [33-37]. In a single-center study of 2,732 patients undergoing CABG, van de Watering *et al. *[38] found no correlation between storage time variables and 30-day mortality in adjusted multivariate analyses. In this study, 30-day mortality was similar in patients receiving exclusively older (mean: 24.3 +/- 3.5 days) or younger RBCs (mean: 12.7 +/- 2.8 days). In a retrospective study of 1,153 cardiac surgery patients, McKenny *et al. *[39] found no association between RBC storage duration and postoperative mortality in a multivariate analysis. In another retrospective, single-center study of 3,597 patients with isolated CABG, van Straten *et al. *[40] found no effect of RBC storage duration on early or late postoperative mortality. In a single-center, retrospective cohort study of 670 patients undergoing CABG and/or valve surgery, Yap and coworkers also found no association between storage duration and mortality [41].

Two retrospective studies examined the association of RBC storage with mortality in critically ill patients. The first was a small cohort of 31 septic patients in which non-survivors received a greater proportion of older RBCs (> 20 days old) compared to survivors, who received a greater proportion of younger RBCs (< 10 days old) [42]. In another retrospective, single-center study of 534 general ICU patients, there was no association between the age of transfused red cells (median of maximum age of RBCs: 23 days) and mortality in multivariate analyses [43].

We found six studies with mortality as an outcome in trauma patients. In a retrospective cohort study of 1,813 severe trauma patients (mean Injury Severity Score (ISS) 26), transfusion of large (but not small) volumes of older blood (more than 14 days old) was associated with an increased risk of death compared to transfusion of the same volumes of younger blood (< 14 days old), suggesting that the age of transfused RBCs could potentiate the already known association between volume of blood transfused and mortality, even though the authors used universal leukoreduction [44]. The same authors reported similar data in another retrospective study with a somewhat higher mortality rate with older blood when patients received more than three RBC units (20.1% in fresh blood vs. 27.0% in the old blood group, *P *= 0.08) [45]. In less severely injured trauma patients (mean ISS 14.4), transfusion of older blood (> 14 days old) was also associated with increased mortality, although to a lesser extent (OR: 1.12, CI: 1.02 to 1.23) [46]. In a smaller retrospective analysis of 202 patients transfused with at least five units of RBCs, Spinella *et al. *[47] found that hospital mortality was higher for patients transfused with older RBCs (maximum RBC age: 28 or more days) than with younger RBCs (maximum RBC age less than 28 days) (26.7% vs. 13.9%, *P *= 0.02). In a multivariate analysis, increased storage time was independently associated with mortality (OR: 4.00; 95% CI: 1.31 to 11.61). Not all studies in trauma patients have reported deleterious effects of RBC storage on mortality. In a retrospective study of 271 trauma patients, Murrell *et al. *[48] found no correlation between the age of blood (expressed as the 'dose of aged blood') and hospital mortality (OR: 1.21, 95% CI: 0.87 to 1.69) after controlling for confounders. In another retrospective cohort of 820 transfused trauma patients, the total number of RBC units but not the number of older (> 14 days old) units transfused was independently associated with increased mortality [49]. These studies were, however, smaller than the previous ones.

Two studies examined the outcomes of medical patients with cardiovascular disease. In a retrospective study of 4,933 patients, Eikelboom *et al. *[50] showed a greater risk of hospital mortality (relative risk 1.48, 95% CI: 1.07 to 2.05) in the highest age quartile of RBCs (31 to 42 days). In a retrospective cohort study of 909 transfused patients following percutaneous coronary intervention, RBC storage duration was associated with 30-day mortality (HR: 1.02 (1.01 to 1.04), *P *= 0.02). Patients receiving only RBCs aged > 28 days had an even higher risk of mortality (HR: 2.49 (1.45 to 4.25), *P *= 0.001) [51]. In a retrospective analysis of 509 patients undergoing liver transplantation, transfusion of more than 10 units of RBCs was associated with increased two-year mortality, but the age of the transfused RBCs was not [52]. In a secondary analysis of a prospective multicenter study on patients with colorectal cancer [53], transfused patients had a shorter survival than the non-transfused patients (3.0 years vs. 4.6 years, *P *= 0.004) but mortality was not significantly different between patients transfused with RBCs aged more or less than 21 days. In a retrospective single-center study on 555 patients undergoing hematopoietic stem cell transplantation, non-relapse 100-day mortality was reduced in the subgroup of patients receiving exclusively old blood (> 14 days old) compared to those receiving fresher (< 14 days old) blood (6% vs. 0% at 100 days, *P *= 0.04) [54].

### Influence on hospital or ICU length of stay

Nine studies evaluated the LOS as a surrogate marker of global morbidity associated with RBC transfusion. Six of the studies included cardiac surgery patients; when controlling for potential confounders, there was no relationship between age of transfused RBCs and postoperative LOS in five of the studies [38,39,41,55,56]. In the only prospective study, there was a significant association between ICU LOS and number of units transfused but not the mean duration of RBC storage [55]. Other retrospective studies yielded similar results. In the study of van De Watering *et al*. mentioned earlier [38], storage time had no independent effect on ICU LOS. In the studies by Yap *et al. *[41] and McKenny *et al. *[39], there was no association between length of storage and ICU LOS. In another retrospective study of 268 patients undergoing CABG, Vamvakas and Carven could not find any association between RBC storage duration and postoperative ICU and hospital LOS in a multivariate analyses also controlling for number of units received [56]. One retrospective study of 444 cardiac surgery patients by Sanders *et al. *[57] yielded different results, with the age of blood a significant but modest predictor of postoperative LOS (OR: 1.05, 95% CI: 1.01 to 1.09). However, this analysis was limited by a number of imbalances between groups.

Two studies evaluated LOS in trauma patients. In a small retrospective study of 86 transfused patients, the number of units of blood older than 14 days was strongly associated with an increased hospital LOS [58], each additional unit older than 14 days old being associated with an increased length of stay of two days. In the study by Murrell and coworkers mentioned earlier, the dose of aged blood was significantly associated with a longer ICU stay (RR 1.15, 95% CI: 1.11 to 1.20) after controlling for confounders [48].

In the study by Kekre and coworkers on patients undergoing hematopoietic stem cell transplantation [54], there was no correlation between age of transfused RBCs and hospital LOS.

### Influence on infections

Eighteen studies reported on the possible influence of age of transfused RBCs on the risk of infection.

Studies performed in cardiac surgery patients generated conflicting results. In the prospective study of Leal-Noval and coworkers [55], there was an independent relation between the oldest RBC unit transfused (but not the mean duration of storage of all the transfused units) and the development of postoperative pneumonia (OR: 1.06, 95% CI: 1.01 to 1.11, *P *= 0.018), but not mediastinitis. In the retrospective study by Koch and coworkers [32], patients transfused with older RBCs (> 14 days old) had significantly higher rates of postoperative sepsis or septicemia (4.0% vs. 2.8%, *P *= 0.01), whereas rates of pneumonia and wound infections were similar. Two other studies reported detrimental effects associated with storage time of non-leukoreduced RBCs. In a retrospective, single center study of 256 cardiac surgery patients, Vamvakas *et al. *[59] reported an independent relationship between the age of transfused RBCs and the incidence of postoperative pneumonia or wound infection after correcting for the number of units transfused. In this study, the risk of pneumonia increased by 1% per day of increase in the mean storage time of transfused red cells (*P *< 0.05). In a multicenter retrospective Danish study on 1,748 patients transfused after CABG, storage time more than 14 days was associated with a greater risk of postoperative wound infections and septicemia (adjusted OR: 2.5, 95% CI: 1.2 to 4.2). The adjusted risk of severe infection increased by 23% for each unit transfused in patients receiving only old RBCs [60]. Other studies found no link between storage time of transfused RBCs and infections. In the smaller cohort of Yap and coworkers [41], no association was found between storage of RBCs and postoperative pneumonia in multivariate analyses. Similar findings were reported in the study by McKenny and coworkers [39].

Two studies were conducted in patients undergoing surgery for colorectal cancer. In a single-center, retrospective study performed between 1980 and 1992 on 290 patients undergoing colorectal resection for adenocarcinoma, age of transfused RBCs was not different in subjects developing postoperative infections and those who did not [61]. In a retrospective analysis of 225 patients undergoing colorectal cancer surgery, Mynster and Nielsen [62] found that a blood storage time exceeding 20 days was an independent risk factor for postoperative infection (wound or intra-abdominal infection, anastomotic leakage, pneumonia, septicemia; OR, 2.35; 95% CI, 1.27 to 4.37; *P *= 0.007).

Five studies were conducted in trauma patients, all suggesting deleterious consequences of storage duration on infectious complications. In the study by Weinberg and coworkers [46] evaluating 1,624 trauma patients, occurrence of pneumonia was directly related to the volume of old blood (> 14 days old) transfused (OR 1.10, CI 1.04 to 1.17), but not young blood (< 14 days old), after multiple adjustments. Also, in a retrospective cohort of 1,183 transfused trauma patients, receipt of exclusively old RBCs (> 14 days) significantly increased the risk of pneumonia (adjusted RR for age, gender, ISS, mechanism of injury, ventilator days and RBC volume 1.42 (1.01 to 2.02)), whereas the transfusion of exclusively young RBCs (< 14 days) or mixed RBCs did not [63]. In a smaller study of 61 massively transfused trauma patients, those who developed major infections had received more units of RBCs greater than 14 or 21 days old. The number of units older than 14 and 21 days was identified as an independent risk factor for major infections in a multivariate analysis [64]. In the previously described study by Hassan and coworkers [49], the number of older (> 14 days old) units of RBCs transfused was a significant risk factor for severe sepsis or septic shock especially when more than seven units were transfused (OR 1.9, 95% CI: 1.1 to 3.4, *P *= 0.03). Recently, in a retrospective study of 196 transfused trauma patients receiving selective digestive tract decontamination, Juffermans *et al*. found a modest association between transfusion of RBCs older than 14 days and occurrence of new infections in multivariate analysis (OR 1.04, 95% CI 1.01 to 1.07) [65].

We identified three studies in septic and/or ICU patients. In a prospective study of 449 medical and surgical ICU patients, Taylor and colleagues [66] found that the number of units of transfused RBCs was associated with an increased risk of nosocomial infection in a multivariate regression analysis, but the maximum age of transfused RBCs was not. In a retrospective study of 134 patients with sepsis, Juffermans *et al. *[67] found a direct relation between the total number of transfused RBCs and the risk of developing secondary infections (OR 1.26, 95% CI 1.09 to 1.45) after controlling for immunosuppression. Storage time was identified as a confounder for the association of RBCs with infection. In another retrospective, single-center study of 534 general ICU patients, Dessertaine *et al. *[43] found no independent association between the age of transfused red cells (median of the maximum RBC ages: 23 days) and the development of nosocomial infection.

The risk of infection was also studied in other populations. In a retrospective analysis of 509 patients undergoing liver transplantation, Dunn *et al. *[52] reported no independent association between the age of transfused RBCs and the incidence of postoperative infections. In a secondary analysis of an RCT on patients undergoing knee arthroplasty, there was no independent association between postoperative wound infection and age of transfused RBCs [68].

### Influence on organ failure (including acute kidney injury and respiratory failure)

We identified 12 studies that addressed the effects of transfused RBCs on multiple organ failure (MOF).

Four studies looked at postoperative acute renal failure in cardiac surgery patients. A retrospective study by Koch and coworkers [32] found an increased risk of MOF (unadjusted comparison 0.7% vs. 0.2%, *P *= 0.007) and renal insufficiency (unadjusted comparison 2.7% vs. 1.6%, *P *= 0.003) associated with transfusion of older blood (> 14 days old). In a multivariate analysis, there was an increased risk of a composite outcome (including MOF and renal failure) associated with transfusion of older blood (adjusted OR: 1.16; 95% CI: 1.01 to 1.33, *P *= 0.03). A smaller study by Sanders and coworkers [57] also showed somewhat higher rates of renal failure among patients transfused with any old blood (12.5% vs. 3.1%, *P *= 0.054) (unadjusted risks) compared to other patients. In a multivariate analysis, every day increase in storage was associated with a 7% increase in risk of new renal complications. These results have been challenged by two other studies that found no association between storage duration and occurrence of postoperative renal failure [39,41].

Two studies included trauma patients. In a small study of 63 matched case-control trauma patients, Zallen and coworkers found that mean age of blood and number of units older than 14 or 21 days were independent risk factors for MOF in a limited multivariate analysis [68]. In a study by Weinberg *et al. *[45] the receipt of old blood (> 14 days old) was associated with acute renal dysfunction (OR 1.18, CI 1.07 to 1.29) after adjustment for age, sex, ISS, thoracic injury and need for mechanical ventilation, whereas the receipt of young blood (< 14 days old) was not.

Some studies focused on the development of respiratory failure, assessed by physiologic and immunologic variables, or the duration of mechanical ventilation (MV). In a randomized, crossover study of 35 healthy volunteers, Weiskopf and coworkers [69] found equivalent mild decrements in pulmonary gas exchange (alveolo-arterial oxygen gradient ((A-a)DO_2_), dead space to tidal volume ratio (V_D_/V_T_)) after isovolemic transfusion of either two units of fresh (1.7 hour) or older (> 21 days) autologous RBCs. No differences between leukoreduced and non-leukoreduced units were noted. Also, in a single-center randomized clinical trial in mechanically ventilated ICU patients, patients randomized to receive exclusively fresh RBCs (*n *= 50 patients, median RBC age: 4.0 days) had similar measures of pulmonary function (changes in PaO_2_/FIO_2 _ratio, changes in V_D_/V_T_, static compliance) when compared to those receiving standard issue RBCs (*n *= 50 patients, median RBC age: 26.5 days) [28]. Eight other observational studies analyzed the duration of MV or the occurrence of ALI. In the study by Koch and coworkers [32] in 6,002 cardiac surgery patients, patients receiving only old RBCs (14 days) had higher rates of MV > 72 hours compared to those transfused with only younger (< 14 days) blood (unadjusted risk 9.7% vs. 5.6%, *P *< 0.001) but no adjusted risk for longer MV duration was calculated. These findings were not found in six other studies [39,41,46,55,56,58]. In a retrospective study on 181 mechanically ventilated ICU patients, Gajic and coworkers [70] found no association between storage variables (mean age or age of the oldest unit transfused) and occurrence of ALI.

### Influence on tissue oxygenation and the microcirculation

Several studies using various devices addressed the question of whether aged RBCs could alter the microcirculation in humans. In a prospective, non-randomized study of 23 septic patients, Marik and Sibbald [71] reported an inverse correlation between the age of transfused RBCs (three units of stored RBCs of varying age) and the maximal change in gastric mucosal pH (pHi) during the subsequent hours (r = -0.71, *P *< 0.001). In a multivariate analysis, age of transfused RBCs was the only predictor of the changes in pHi, suggesting that older, less deformable RBCs could promote mucosal ischemia. These data were, however, challenged by later studies. In particular, Fernandes and colleagues [72] compared 15 anemic septic patients randomly assigned to receive either one RBC unit (mean storage time: 12.8 ± 8.1 days) or 500 mL of a 5% albumin solution. There was no increase in oxygen delivery or consumption following RBC transfusion (but the baseline hemoglobin concentration was relatively high) and no correlation between the age of transfused RBCs and the pHi. Likewise, in a randomized, double-blinded study on 22 anemic ICU patients, Walsh and coworkers [73] reported no difference in pHi or gastric to arterial PCO_2 _gap with transfusions of two units of leukoreduced RBCs stored for less than five days (median: 2 days) or more than 20 days (median: 28 days). Comparisons of this work with the previous study by Marik and Sibbald [71] is difficult because of differences in patient populations and the use or not of leukodepleted blood [74]. Sakr and coworkers [75] performed a prospective study evaluating transfusion-induced changes in the sublingual microcirculation of 35 septic patients (using an orthogonal polarization spectral imaging technique). Oxygen uptake and microcirculatory variables were globally unaltered after the administration of one or two units of leukoreduced RBCs (median age: 24 days; IQR: 12 to 28 days); considerable interindividual variability in sublingual capillary perfusion was noted among these patients and no correlation between the storage time and the changes in capillary perfusion was found [75].

In another small randomized trial on 20 hematology outpatients transfused with three units of either young (median: 7 days) or older blood (median: 23 days), viscosity and perfused vessel density in sublingual microcirculation (assessed with sidestream dark field imaging - SDF) increased after transfusion, but this increase was similar in both RBC age groups [76]. Interestingly, the aggregability index was also increased following RBC transfusion whereas the RBC deformability index was unchanged, with no differences between older and younger blood groups. Leal-Noval and coworkers [77] assessed the effects of transfusion of leukoreduced RBCs on cerebral tissue oxygenation (PtiO_2_) in patients with severe traumatic brain injury divided into four quartiles according to length of storage of transfused RBCs (< 10 days, *n *= 18; 10 to 14 days, *n *= 15; 15 to 19 days, *n *= 17; > 19 days, *n *= 16). Transfusion of RBCs stored for less than 19 days increased PtiO_2 _for up to six hours, whereas transfusion of RBCs aged more than 19 days failed to do so. There was a trend toward an inverse relationship between storage time and the relative increment in PtiO_2 _from the baseline value, but there were concerns about methodological limitations in this study [78]. Kiraly and coworkers [79] performed a prospective, non-randomized study in 32 hemodynamically stable, non-septic, ICU trauma patients requiring a transfusion. Patients transfused with 'old' red cells (> 21 days old, *n *= 17 patients) had a significant decrease in the area under the curve (AUC) of tissue oxygen saturation (StO_2_) as measured by near-infrared spectroscopy (NIRS [80]), whereas patients receiving 'new' red cells (< 21 days, *n *= 15 patients) and a control, non-transfused group had a stable StO_2 _AUC. Moreover, a slightly negative correlation between the age of the oldest unit transfused and the changes in oxygenation was noted (r^2 ^= 0.25). In a prospective study on 44 hemodynamically stable ICU patients also monitored with NIRS, Creteur and coworkers [81] found no association between RBC storage time (median: 18 days; IQR: 11 to 27 days) and oxygenation variables (changes in the StO_2 _upslope, changes in NIR oxygen consumption). Also, in a randomized crossover study in nine healthy volunteers, reactive hyperemia index (measured by peripheral arterial tonometry and reflecting NO bioavailability and endothelial function) was unchanged after transfusion of one unit of 40-day stored autologous blood compared with transfusion of three-day stored blood [82]. Another recent study on eight healthy volunteers receiving sequentially one unit of seven-day AS-3 stored and one unit of 42-day stored autologous blood found no effect of storage duration on oxygenation variables (tissue oxygen saturation - brain and thenar eminence, and sublingual microcirculatory flow index) [83]. Recently, Kopterides *et al. *[84] reported no relationship between age of transfused RBCs (median: 16 days) and post-transfusional change in lactate/pyruvate ratio (microdialysis assessment) in 37 ICU patients with sepsis.

### Other outcomes

a) Thrombotic events

In a retrospective study of 202 trauma patients, Spinella and coworkers found an association between maximum age of transfused RBCs (> 21 or 28 days) and the occurrence of deep vein thrombosis (DVT) but no multivariate analysis was performed to confirm this observation [47]. Moreover, these results were challenged by a prospective study of 261 medico-surgical ICU patients in which there was no association between the age of transfused RBCs and the occurrence of DVT [85]. Leal-Noval and coworkers found no association between duration of storage of transfused RBCs and postoperative myocardial infarction [55].

b) Vasospasm after subarachnoid hemorrhage

In a retrospective, single-center study of 119 patients transfused after SAH, Naidech and coworkers could not find any association between age of RBCs and poor outcomes (vasospasm, cerebral infarction, dependence or mortality at three months) [86].

c) Bleeding

One may hypothesize that stored blood (especially in the absence of leukoreduction) could increase bleeding through an altered viability of platelets and a decrease in coagulation factors, such as factor VIII. In patients undergoing CABG surgery, Wasser and coworkers [87] found no differences in postoperative bleeding, coagulation tests, or transfusion requirements between the study group (two units of fresh blood followed by stored RBCs (aged from two to five days) if required) and the control group (stored blood only), except for a subgroup of patients with thrombocytopenia.

d) Cognitive dysfunction

In a randomized crossover study on healthy volunteers submitted to acute normovolemic hemodilution (target hemoglobin 5 g/dl), Weiskopf *et al. *[88] found that reversal of anemia-induced cognitive dysfunction was similar after transfusion of two units of fresh (storage less than five hours) or stored (three weeks storage) RBCs.

e) Cancer recurrence

Immunomodulation associated with RBC transfusion may influence cancer recurrence after surgery. In a retrospective study of 740 patients with colorectal cancer, Mynster and coworkers [53] found a higher recurrence rate of cancer in patients who received a transfusion of RBCs stored < 21 days (HR: 1.5; 95% CI: 1.04 to 2.18, *P *= 0.03 for colorectal cancer) than in patients who received blood stored ≥ 21 days. Another retrospective study of 316 patients undergoing surgery for prostate cancer found no association between the age of transfused allogeneic RBCs and the five-year cancer recurrence defined by prostate specific antigen levels [89].

## Discussion

It is interesting to note the quasi-exponential growth in the number of publications related to the impact of RBC storage since 1989, with 50% of the studies published during or after 2009. We identified 55 studies that reported an association between RBC storage and outcomes in adult patients. Of these, 26 studies (47%) suggested detrimental effects of RBC storage on any clinical endpoint, whereas the remainder (53%) did not.

Comparison among studies is difficult because of the high degree of heterogeneity. First, studies included different patient populations (cardiac surgery, trauma, sepsis, cancer patients) with different baseline risks. Interestingly, 91% of the studies on trauma patients showed a deleterious impact of older RBCs on any endpoint, whereas only 45% of studies in cardiac surgery patients and 36% of studies in ICU patients suggested such an association. It is intriguing to consider that trauma patients may be more sensitive to the age of transfused RBCs than other patients [90]. Second, studies also included different mortality and morbidity outcomes and the criteria for organ dysfunction varied. Third, the way in which the age of transfused RBCs was reported also varied (mean age of all RBCs transfused, maximum age of RBCs, mean of the two oldest units, proportion of RBCs older than a given number of days,...), each analysis having its own advantages and limitations. Studies that report the mean or median age of all units transfused assume that fresher units offset the deleterious effect of older blood but this may not be true. Dichotomization of RBC age below or above a given time based on more or less proven pathophysiological evidence may also be problematic [18,91]. Recently, in a simulation-based analysis of four RCTs (three currently ongoing) that used distinct categorization of RBC storage, the type of temporal pattern assumed for the RBC storage lesion (smooth or sharp sigmoid curve centered on 7, 21 or 35 days; linear relation) had a profound impact on the statistical power of the trials for various outcomes, such that it was below 80% in numerous cases [91]; in particular, none of the studies had sufficient power to detect harmful consequences of RBC storage lesions if they were assumed to occur during the last week of storage. One must take into account the fact that older units are more often transfused during massive transfusions, which are obviously associated with worse outcomes [18]. Fourth, the blood preparation, including leukoreduction and use of additive solutions, varied among the studies; these practices have evolved over time and place and may influence the results. We found only 17 studies (35% of all studies) that reported use of exclusively leukoreduced RBCs, 10 studies (20%) reporting use of exclusively non-leukoreduced units and 11 studies (22%) reported use of a mix of leukoreduced and non-leukoreduced RBCs.

Most of the studies were retrospective with associated limitations. Control for potential confounders is particularly important. Problems with bias (recently reviewed and listed in [18]) are well illustrated by the phenomenon of confounding by indication, occurring when the indication for a treatment causes the outcome studied [92]. The most notable example of this effect concerns sicker patients who have a poorer prognosis and are also prone to receive more blood transfusions, leading, if no correction is applied, to the spurious association between number of blood transfusions and poorer outcomes. Another, more subtle problem is that patients who need multiple transfusions are, in general, more likely to receive some older blood [38,92]. Transfusion thresholds, which have evolved over time and may vary between centers, may also play a role because they may modify the global exposure of a patient population to blood transfusions. Also, temporality of the association between transfusion and outcome is not always clear. Statistical methods, like multivariate analyses, are required to correct for some biases (for example, correction for the total number of units transfused, which is one of the most important biases in the transfusion literature). In some studies, there was poor control for the number of transfused RBCs or imbalances in ABO groups [32,93] and possibly other unknown biases. The small number of patients included in many studies may also be of concern: 'negativity' of small studies should not be interpreted as proof for absence of effect [18].

Unfortunately, performing a quantitative meta-analysis is complicated by the significant clinical heterogeneity between studies, even if tests for statistical heterogeneity are passed. Moreover, meta-analyses do not improve the quality of individual studies [94], a major concern in our systematic review, which identified numerous biased studies. For these reasons we chose not to perform a meta-analysis. Nevertheless, some have been attempted [20-22], resulting, not surprisingly, in conflicting results. The most recent meta-analysis by Wang and coworkers showed detrimental effects of RBC storage on mortality (pooled OR 1.16; 95% CI 1.07 to 1.24, *P *= 0.0001), pneumonia (pooled OR 1.17; 95% CI 1.08 to 1.27, *P *= 0.0001) and multiple organ dysfunction syndrome (pooled OR 2.26; 95% CI 1.56 to 3.25, *P *< 0.0001); in this study, 97 patients (95% CI 63 to 199) was cited as the number to treat with exclusively fresh blood to save one life [20]. Another meta-analysis by Vamvakas that included only studies reporting adjusted results for mortality, found that the age of blood was not associated with a higher risk of mortality [22].

Transfusion policies in some centers recommend use of fresh RBCs for special populations, such as fetuses (intrauterine transfusions) or neonates, although the evidence for this approach is poor [95]. If one could choose, one would prefer fresher blood, although storage for a few days at 4°C may be beneficial to reduce the risk of some cell-associated viruses and other pathogens that cannot survive for long in these conditions [95]. Moreover, the delivery of fresher blood may be associated with major organizational problems although some recent pilot trials suggest the feasibility of such an approach [96,97]. Some would argue that the best way to resolve this problematic issue with adequate control for known and unknown biases may be achieved by large RCTs. It is intriguing to note that among eight randomized trials already published (without pilot trials) [28,69,72,73,76,82,87,88], none demonstrated detrimental effects of RBC storage duration on outcomes, although the studies were small and mostly reported physiological outcomes. Several large randomized studies are ongoing (notably ABLE [98] and RECESS [99]) and their investigators have had to face various obstacles. First, as the differences in clinically relevant outcomes (such as mortality) are (if present) expected to be low, large numbers of patients are required to detect these differences. Second, these trials must overcome logistic problems so that the blood banks can appropriately deliver exclusively young or older RBC units (compared to an *a priori *defined threshold), as illustrated in several studies [96,100-102]. Third, even if the duration of storage is still within acceptable limits, administration of exclusively older RBCs to patients may raise ethical concerns. Use of standard RBCs instead of exclusively old RBCs may overcome this issue (for example, in the ABLE study [98]).

Our study has several strengths and limitations. First, we considered clinically relevant outcomes, which we discussed separately and were the basis for the study classification. Studies reporting biological outcomes (hemolysis [103], survival of RBCs after transfusion [104], hematocrit response [105] or mitogenic activity of plasma after transfusion [106]), or evolution of clinical scores after RBC administration [107], were not considered. Pediatric studies were also not included, because newborns and children may represent a particular population with specific pathologies. Also, we did not consider feasibility studies (pilot trials before large RCTs) [96,97,101], which were not primarily powered to assess the clinical relevancy of RBC storage duration. Finally, we chose to refrain from performing a quantitative meta-analysis because of the high number of biased studies, which could have led to erroneous conclusions.

In conclusion, our systematic review indicates that the influence of RBC age on various outcomes is equivocal. The quality of the evidence is currently too poor to make recommendations to change current transfusion practice. Our observations are reassuring, because major differences would have serious consequences for the organization of blood banks [108].

## Key messages

• The duration of red blood cell (RBC) storage before transfusion may alter RBC function and, hence, potentially influence the incidence of complications or even mortality, but this is controversial.

• Fifty-five studies were identified that had reported the effects of age of transfused RBCs on mortality or morbidity in adult patients, but the marked heterogeneity among studies prevented a formal meta-analysis.

• Half of the studies showed no deleterious effects of aged compared to fresh blood on any endpoint.

• This systematic review found no definitive argument to support the superiority of fresh over older RBCs for transfusion.

## Abbreviations

A-aDO_2_: alveolo-arterial oxygen gradient; ALI: acute lung injury; ARDS: acute respiratory distress syndrome; AUC: area under the curve; CABG: coronary artery bypass grafting; CI: confidence interval; DVT: deep vein thrombosis; HR: hazard ratio; ICU: intensive care unit; IQR: interquartile range; LOS: length of stay; MOF: multiple organ failure; MV: mechanical ventilation; NIRS: near-infrared spectroscopy; OR: odds ratio; pHi: gastric mucosal pH; PtiO_2_: cerebral tissue oxygenation; RBC: red blood cell; RCT: randomized controlled trial; RR: relative risk; SAH: subarachnoid hemorrhage; SD: standard deviation; SDF: sidestream dark field; StO_2_: tissue oxygen saturation; V_D_: dead space; V_T_: tidal volume.

## Competing interests

The authors have no competing interests to declare in relation to this article

## Authors' contributions

CL and JLV designed the study. CL performed the literature search and drafted the manuscript. JLV critically revised the text. Both authors read and approved the final manuscript.
